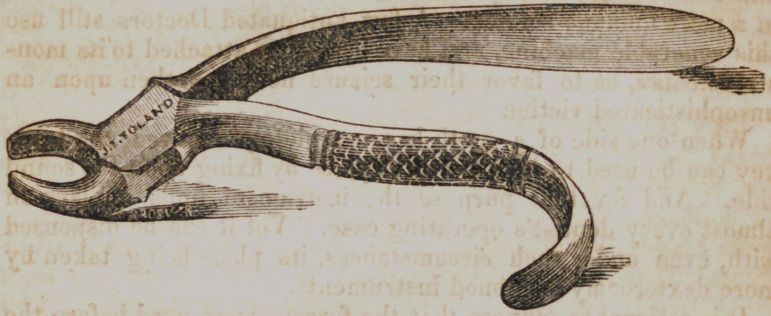# Extracting Teeth

**Published:** 1858-04

**Authors:** A. J. Howe


### EXTRACTION OF TEETH.

BY PROF. A. J. HOWE.

In cities and large towns the extraction of teeth is confined—and properly too—to the dental profession, and the physician and surgeon seldom keep themselves supplied with instruments necessary to perform the operation. The dentists having the business exclusively, fit up their operating cases with every variety of instrument that might possibly be needed in the complications of an extensive business. They expend hundreds of dollars in their "out-fit," and constantly replenish with every valuable accession to their enterprising and progressive science. Were there members of the dental profession in every town and village in the land, an article on the subject of tooth extracting would not be appropriate for a Medical Journal. But it would be suggested that members of the medical fraternity turn over to the dentist such business as legitimately belongs to his profession.

The people in rural districts and small villages are too far removed from a dentist to employ him, except one of the itinerant variety who cares more for dimes than a valuable reputation; consequently the whole dental profession is regarded by them as a superfluity, and one that could be as easily dispensed with, as the clan of jewel-vending Jews.

It is well for all physicians to understand so much of dental science as to be able to treat successfully odontalgia, and to extract teeth when patients from illness, or distance from a dentist's office, are unable to obtain the services of one of that profession. In order to execute in a workmanlike manner this branch of the dental art, the physician must be supplied with a few instruments, such as a flexible probe for examining carious cavities, and forceps for removing the several teeth, and their fangs.

The cases of odontalgia are too various to demand a single course of treatment. A general neuralgic state of the system may produce such a painful condition of the teeth and jaws as to incite the patient to seek relief in extraction. But no judicious physician will so far yield to the entreaties of a patient as to remove a sound tooth simply because it aches. Pregnant females often suffer from pain in the teeth, and relief would not follow the extraction of every tooth in the jaws. The most common cause of toothache is exposure of the nerve from caries. The nerve is irritated by cold currents of air, acid fluids, and the pressure of food. Teeth affected in this way—if the cavity be not too extensive—should be "filled." Yet there may not be any competent dentist in the vicinity, when it will become necessary to perform the operation of "extraction," or apply some local remedy to allay the pain. Before having recourse to professional advice and aid, patients commonly resort to the local use of camphor, paragoric, laudanum, and other popular remedies. The physician when consulted, for a time may continue the use of local anodynes, but permanent relief not following such treatment, he generally resorts to extracting instruments. It may be better, however, in cases where the patient can at some subsequent period obtain the services of a dentist to have a filling inserted, to destroy the nerve. This may be done in two principal ways. Firstly by forcing to the bottom of the fang, along the canal which contains the nerve and blood-vessels distributed to the pulp of the tooth, a flexible steel wire the size of a bristle, and withdrawing it with a twisting motion. This operation removes the nerve, and of course the source of irritation and pain. The introduction of the wire is attended with keen, momentary suffering, and few patients knowing the nature of the operation can calmly submit to it. The pain, however, is not so severe and lasting as that attending extraction. Nearly all dentists of the present day extract the nerve and force the filling to the bottom of the fang. And when well done, it is the most efficient method of precluding any further trouble on the part of that tooth.

The other method of destroying the nerve consists in the direct application to the pulp, of a preparation of arsenic. The formula as used by most dentists for this purpose, is as follows:

℞.—Arsenious acid, grs. iv.
Morphiæ Sulph., grs. ij.
Creosote, q. s.
Misce, fiat massa seu pasta.

This preparation used in minute quantities, sufficient only to destroy the vitality of the nerve and blood-vessels of the cavity, is safe and efficient. Before using it the carious cavity should be well cleansed with a probe that the paste may be placed in immediate contact with the pulp or nerve. A dossil of lint the size of a pin's head when compressed, smeared with the paste, is then to be pressed into the cavity with a probe, and confined there with a layer of wax. This should be kept in place for thirty-six hours, when it is to be removed to prevent the arsenic from penetrating beyond the extremity of the fang, and irritating the external or peridental membrane of the tooth within the alveoli. Immediate relief generally follows the application of the paste, and the nerve is so thoroughly destroyed, that it rarely serves as a medium of sensibility. If the peridental covering be not affected by the arsenic of the paste, the tooth will retain its vitality and to a great extent be preserved from discoloration and decay. The tooth can be filled at any time subsequently, yet the sooner the better. If the fang be not filled, there is constant danger of inflammation and suppuration about its extremity which commonly can not be relieved without a removal of the entire tooth. The pain that often attends a carious tooth, is quite as excruciating as any mortals have to endure. And extraction is accompanied with as keen pangs as the amputation of a limb. But as the operation is brief, and the relief so certain, few persons will endure toothache a great length of time, without attempting to rally courage enough to meet the extracting instruments.

The object of this paper is more to demonstrate the means of extracting teeth, and the most limited number of instruments that can be employed and yet be sufficient, than to detail the various means of arresting tooth-ache by other agencies. And for this purpose several diagrams have been gotten up to illustrate the very few forceps that will answer to extract the different teeth and fangs. A fewer number could not furnish the variety of form that is actually demanded for the extraction of the several teeth without danger of breaking, and other accidents; and a greater number of forceps would require more expense than a physician is ordinarily willing to incur for this branch of his professional business. Were a practical dentist asked to select from a stock of dental goods the smallest number of forceps a physician would require in the general practice of tooth extracting, he certainly would not name less than four pairs; and even that small number would not be suggested without hesitation and forethought.

This pair of forceps is needed to extract fangs anywhere in the jaws, and may serve to remove the lower incisors, and the smallest teeth of children. This instrument should be made of good stock, and so tempered as not to bend or break under the force ordinarily applied to it. The inner surface of the beaks are made slightly concave to clasp the convexity of a fang or the neck of a small tooth, and slender enough to easily slide between the gum and tooth, even down to the alveolar borders. No gum lancet need be used to cut around a tooth before it be extracted, as the gums do not ordinarily adhere to the teeth. To use a lancet for incising a gum before applying a forcep is a barbarous practice; and it requires about as much resolution on the part of a patient to have the gums cut, as it does to meet the forceps.

When fangs are to be extracted, it may be well sometimes to cut away the gums, that the forcep blades may grasp the thin alveolar borders which are to be cut through with the forceps before the fang is reached. The lesion done to the alveoli by such an operation, is not serious in its results. The injury to the bone and soft tissues in such cases granulates and cicatrizes about the same time, and the process of recovery is not much prolonged by the complication.

This cut represents a pair of forceps, whose strength and beaks are adapted to the extraction of the superior incisors, the cuspidati and bicuspidati teeth, as well as the larger teeth of children. The variety of teeth to be removed by this instrument renders it impossible for the blades to fit accurately all of them. Consequently they are to be used with more care than is ordinarily exercised in the application of forceps. The breaking of a tooth in an attempt at extraction, is particularly to be avoided. If the inside of the beaks be too concave, the instruments will break off the neck of the tooth; and if they be too straight, they will crush the crown of a frail one. These accidents often occur from badly fitting instruments.

The instrument here figured is adapted to the extraction of the inferior molars, including the wisdom teeth of the lower jaw. In place of this pair, a practical dentist is accustomed to use at least three pairs. One instrument, however, fashioned like the one above represented, in good hands, will successfully remove all the molars of the inferior maxilla. To use the instrument advantageously, the operator should stand on the right of the patient, behind his arm, and facing his side. The patient's head thrown back to expose to view the teeth, is to be held firmly with the left *arm*, while the left *hand* steadies the jaw to prevent fracture or dislocation. It is essential that the operator stand upon a stool unless the patient be placed upon a low seat. Without this precaution many teeth will be broken that might be entirely removed by the first attempt. The blades of the forceps must be well arched on the inside, or the crowns of decayed teeth will be crumbled by their pressure. In the successful construction of this, as well as other dental instruments, the cutler should have been a practical dentist.

None of the instruments previously mentioned in this article, are fit to extract upper molars, consequeutly this one remains to be described. It is a more powerful forcep than either of the others. It has a curve in it near the joint to let it back into the mouth, and at the same time to give the operator control over these triple fanged, firmly set teeth. That the hand may exert more power upon the handles of this forcep, a curve is made in one of them to wind around the little finger. An instrument like the one in the diagram is well adapted to the extraction of the superior molars, and the upper wisdom teeth. For the successful use of these forceps, the patient should be placed on a low seat, or the operator should be elevated. It needs a firm grasp to prevent the backs of the instrument from slipping on the neck of the tooth. To cant the tooth powerfully to one side and then the other, is necessary to remove it easily and with as as little pain as possible. But to repeat the cantings rapidly by rotating the hand is not good practice. Each lateral movement of the tooth pinches the peridental membrane, and elicits as much pain as a cant heavy enough to loosen a fang.

With four pairs of forceps similar to those represented by the diagrams, a physician may extract any tooth that demands the operation. These four pairs, constituting what is called the *physician's set*, were devised by us, and are sold by J. T. Toland, of this city.*

At the present day, scarcely any thing need be said concern-

*The "Physicians set," is sold for six and nine dollars, according to the quality of the finishing polish.

ing the *turnkey* which for centuries served as the only tooth extracting instrument. Almost every adult can vividly call to mind, without the assistance of a diagram, the outline of this old fashioned tormenter. It has been suggested that it was first introduced into practice by a cruelly ingenious inquisitor; and that it caused many a heretic to recant before it was used in a more humane service. A few antiquated Doctors still use this venerable machine, and have become so attached to its monstrous *claws*, as to favor their seizure now and then upon an unsophisticated victim.

When one side of a tooth has completely decayed, the turnkey can be used to *turn* it out of place by fixing upon the sound side. And for that purpose the instrument finds a place in almost every dentist's operating case. Yet it can be dispensed with, even under such circumstances, its place being taken by more dexterously fashioned instruments.

It is rational to suppose that the forceps were used before the introduction of the turnkey. In the temple of Apollo which was built centuries before the Christian era, has been found a leaden forcep, which was undoubtedly the model of an instrument used in precisely the same manner that extracting forceps now are. The ancient model is uncouth but in all likelihood, was suggested by the early use of the fingers in the removal of teeth that barely adhered to the gums. It required a modern mechanic to construct forceps, whose form and temper should be adapted to the extraction of every variety of teeth. Much credit is due the enlightened profession that has carried within the space of twenty-five or thirty years, to the perfection of a science, an art that was recently among the rudest. It only remains for the more philanthropic in the profession to diffuse such knowledge among the people, as will enable them to better preserve *from the need* of the operating dentist, those highly useful and ornamental organs, the teeth.

The above, we copy from the *College Journal of Medical Science*, conducted by the Faculty of the Eclectic College of Medicine.

We have only to add the prices, as follows:

Oval Jointed Forceps, $1,50 each; $6,00 a set;

Octagon Jointed Forceps, crocus polish, $2,25 each; $9,00 a set.

JNO. T. TOLAND.

38 West Fourth Street.

## Figures and Tables

**Figure f1:**
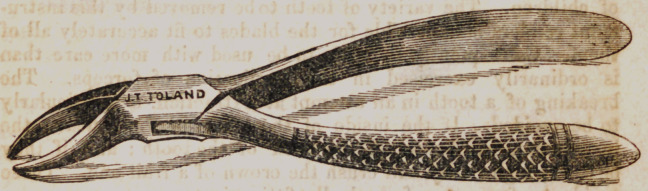


**Figure f2:**
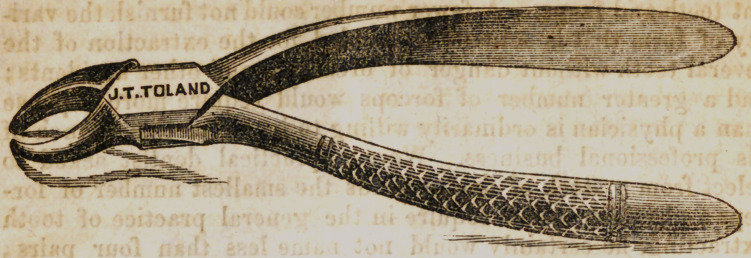


**Figure f3:**
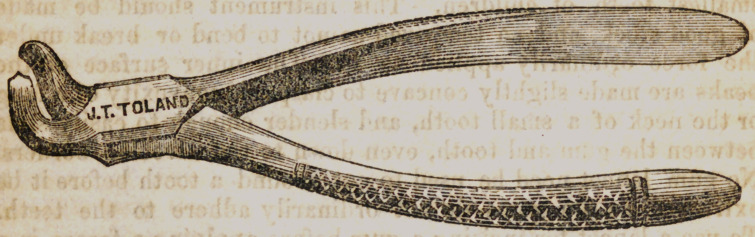


**Figure f4:**